# Preparation and Characterization of Esterified Bamboo Flour by an In Situ Solid Phase Method

**DOI:** 10.3390/polym10080920

**Published:** 2018-08-16

**Authors:** Yaqi Geng, Xiaohan Pei, Xiaoyu He, Ping Li, Yiqiang Wu, Yingfeng Zuo

**Affiliations:** 1 College of Materials Science and Engineering, Central South University of Forestry and Technology, Changsha 410004, China; Joyg0428@163.com (Y.G.); pxh2376929985@outlook.com (X.P.); h2221810261@163.com (X.H.); pzxiaomei@163.com (P.L.); wuyq0506@126.com (Y.W.); 2 Hunan Provincial Collaborative Innovation Center for High-efficiency Utilization of Wood and Bamboo Resources, Central South University of Forestry and Technology, Changsha 410004, China

**Keywords:** bamboo flour, in situ solid phase method, esterification modification, substituting degree, hydrophobicity

## Abstract

Bamboo plastic composites have become a hot research topic and a key focus of research. However, many strong, polar, hydrophilic hydroxyl groups in bamboo flour (BF) results in poor interfacial compatibility between BF and hydrophobic polymers. Maleic anhydride-esterified (MAH-e-BF) and lactic acid-esterified bamboo flour (LA-e-BF) were prepared while using an in situ solid-phase esterification method with BF as the raw material and maleic anhydride or lactic acid as the esterifying agent. Fourier transform infrared spectroscopy results confirmed that BF esterification with maleic anhydride and lactic acid was successful, with the esterification degrees of MAH-e-BF and LA-e-BF at 21.04 ± 0.23% and 14.28 ± 0.17%, respectively. Esterified BF was characterized by scanning electron microscopy, contact angle testing, X-ray diffractometry, and thermogravimetric analysis. The results demonstrated that esterified BF surfaces were covered with graft polymer and the surface roughness and bonding degree of MAH-e-BF clearly larger than those of LA-e-BF. The hydrophobicity of esterified BF was significantly higher than BF and the hydrophobicity of MAH-e-BF was better than LA-e-BF. The crystalline structure of esterified BF showed some damage, while MAH-e-BF exhibited a greater decrease in crystallinity than LA-e-BF. Overall, the esterification reaction improved BF thermoplasticity, with the thermoplasticity of MAH-e-BF appearing to be better than LA-e-BF.

## 1. Introduction

In China, bamboo is a rich resource, having the benefit of a short growth cycle, and the research and utilization of bamboo have attracted much attention [1,2]. Various kinds of bamboo wood-based panels, active carbon, and fuel have been successfully developed [3,4]. However, these traditional processing methods have low utilization rates, high energy consumption, serious pollution issues, lack of effective utilization, single performance uses, and low added value. If bamboo leftovers from these processes and bamboo plastic composite polymer mixed preparations can be used for packaging, construction, and even used in cars, high-speed rail, aircraft, floors, and interiors, it would not only effectively improve the utilization rate of bamboo timber and produce added value, but it would also promote the development of a circular economy and help to maintain an ecological balance [5,6].

However, there are many strong, polar, hydrophilic hydroxyl groups in bamboo, which are incompatible with polymer interfaces and directly affect the thickness, morphology, structure, and dispersion uniformity of the material, leading to the deterioration of material properties. Therefore, replacing hydrophilic -OH by hydrophobic groups is an effective way to improve the hydrophobic properties of bamboo. The methods for modifying bamboo hydrophobicity, while using existing technology, include bamboo fiber steam explosion [7,8] and electron beam irradiation. These physical methods can improve the mechanical interlocking forces of bamboo flour (BF) and polymer composites to a certain extent, but they cannot form a strong chemical bond between the fiber and resin matrix. For this reason, researchers have used chemical methods to modify these plant fibers. Bledzki has pointed out that a fiber-coupling agent treatment can change fiber wettability and improve the interfacial compatibility of composite materials [9]. However, this method generally requires modification by a coupling agent in an organic solvent, which increases production costs and also has potential to damage the environment. Also, Lee has studied maleic anhydride (MAH)-esterified bamboo fiber compounded into a composite material, which improved the interfacial compatibility [10]. In addition, many studies have used esterification reactions to modify BF and achieve good results [11,12].

**Figure 1:**
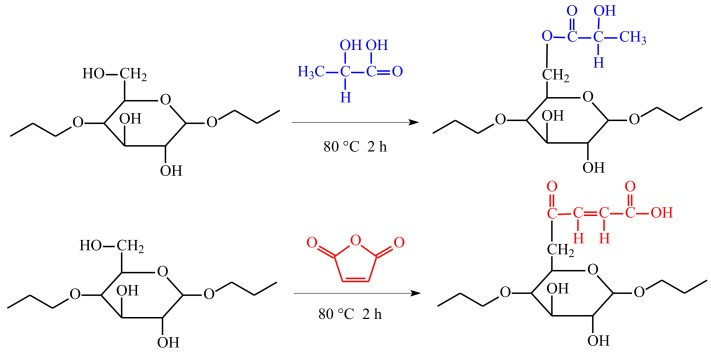
Reaction mechanism of esterified bamboo flour (BF).

Chemical modification of the plastic matrix has been shown to form a molecular/structural bridge between the fibers and plastic, which is conducive to enhancing the bonding between interfaces. At present, the most commonly used esterification methods include an aqueous phase, organic solvent, and reactive extrusion methods. The water phase method is a uniform reaction without organic solvent, because the reaction is a heterogeneous reaction between the solid particles of BF and the liquid, the affinity and forces between the two reactants are not high and the hydrolysis of carboxylic acid is a side reaction. The organic solvent reaction is uniform, but the degree of substitution is not high and the reaction has environmental pollution problems. The reaction extrusion method needs plasticizer, which destroys the BF particle structure and restricts the range of BF applications. In view of this, a solid-phase esterification method in situ was used for modifying bamboo. Lactic acid (LA) and MAH belong to two carboxylic acids, and their use to modify BF can improve its crosslinked structure. In experiments here, BF was mixed with LA or MAH in an airtight reactor and the solid phase esterification reaction carried out under certain pressure and temperature conditions (Figure 1). When compared with the solution method and melting method, solid phase esterification exhibited the following advantages: (1) the reaction temperature was significantly reduced and by-products and degradation reactions also reduced; (2) the monomer concentration was large and fully reacted, the reaction was favored, and the reaction efficiency was high; (3) the condensation reaction and ring-opening reaction was stable and high pressure was not required; and, (4) the reaction system did not require organic solvents. This was overall an environmentally-friendly condensation process. This in situ solid-phase esterification of BF was found to be a highly efficient and environmentally-friendly process.

## 2. Materials and Methods

### 2.1. Materials

The BF that was used in this study was supplied by Hunan Taohuajiang Industrial Co., Ltd. (Yiyang, China) and was used as received. LA (AR) was obtained from Chongqing East Sichuan Chemical Co., Ltd. (Chongqing, China). MAH (AR) was purchased from Tianjin Kemiou Chemical Reagent Co., Ltd. (Tianjin, China). Acetone (AR) was obtained from Hunan Normal University Chemical Reagent Factory (Changsha, China). Sodium hydroxide (AR) was obtained from Xilong Chemical Co., Ltd. (Guangzhou, China). Ethanol (99.7%, AR) was obtained from Anhui Ante Food Co., Ltd. (Suzhou, China). Ultra pure water was obtained in the laboratory.

### 2.2. Preparation of Esterified BF

**Figure 2:**
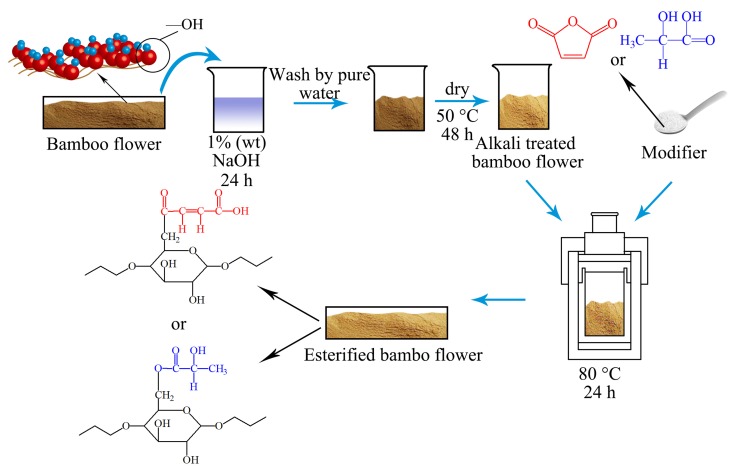
Preparation process of esterified BF.

A select quantity of BF was treated with 1 wt % NaOH solution for 24 h, washed repeatedly with tap water till the wash water pH was neutral and then dried in an oven of 60 °C to a constant weight (Figure 2). Next, 30 g of the alkali-treated BF (dry base) was mixed with 4.5 g of LA or with 4.5 g of MAH and then placed in a hydrothermal reaction kettle at 80 °C for 2 h. The reaction products were cooled to room temperature, a select quantity of acetone added, stirred for a while, and then the solvents removed by rotary evaporation. Finally, the product was washed three times with acetone and then placed into a 50 °C oven and dried till constant weight was achieved.

### 2.3. Properties and Characterization

#### 2.3.1. Fourier-Transform Infrared Spectroscopic Analysis

Chemical changes in esterified BF after esterification were characterized while using Fourier-transform infrared spectroscopy (FT-IR) of samples that were tabletted with KBr and (IRAffinity-1, Shimadzu Corp., Kyoto, Japan). To remove moisture completely, native and esterified BF were further dried in a muffle oven at 50 °C for 48 h. Samples for testing were obtained by grinding material fully with a weight ratio of sample/KBr of 1/100. FT-IR curves of samples were obtained in a range of 400–4000 cm^−1^.

#### 2.3.2. Determination of Esterification Degree

Esterification degree of esterified bamboo flour was tested by the saponification principle. The grafting efficiency was calculated while using a previous published procedure [13,14]. First, 1.00 g of dry esterified BF was weighed and placed in a 250 mL conical flask. Next, 10 mL of 75% ethanol solution in deionized water was added, followed by the addition of 10 mL of 0.5 M aqueous sodium hydroxide solution. The stoppered conical flask was agitated, warmed to 30 °C, and stirred for 1 h. Excess alkali was then neutralized with a standard 0.5 M aqueous hydrochloric acid solution. A blank titration was performed using native BF and the degree of esterification (DS) being calculated as follows.

(1) $$W_{}=\frac{Mc(V_{0}-V_{1})}{1000\times nm}\times 100\%\;$$

(2) $$GR=\frac{162W_{}}{M\times (100-W_{})}\times 100\%\;$$

Where *W* is the substituent group content, %; *M* the esterifying agent molecular weight, g; *c* the aqueous hydrochloric acid solution concentration, M; *V_0_* the aqueous hydrochloric acid solution volume consumed by the blank sample, mL; *V_1_* is the aqueous hydrochloric acid solution volume consumed by the esterified BF sample, mL; *n* the number of hydrophobic groups from the grafted monomer; and, *m* the sample mass, g.

#### 2.3.3. Determination of Water Absorption

To determine the effects of esterification on BF hydrophobicity, 2.0 g of native BF and esterified BF (dry base) were separately placed in glass dishes that contained a set amount of water. Over the test period, the sample weights were measured every 24 h and water absorption was calculated, as follows.

(3) $$\begin{array}{l} \mathrm{water}\;\mathrm{absorption}=\frac{W_{t}-W_{0}}{W_{0}}\times 100\% \end{array}\;$$

where *W_t_* is the sample weight after water absorption for *t* h and *W_0_* the sample weight when it reached a constant dry weight.

#### 2.3.4. Contact Angle Measurements

A set amount of native and esterified BF were weighed and pressed into pie-shaped samples 1.5 cm in diameter using a press machine with a pressure of 20 MPa. An optical contact angle measurement instrument (OCA20, DataPhysics Instruments GmbH, Filderstadt, Germany) was used to measure the sample contact angle, while using distilled water as the test solution. For each measurement, a 4-uL drop of water was placed on a test sample using a microsyringe and the contact angle values measured to within 1°.

#### 2.3.5. Scanning Electron Microscopic Analysis

The morphologies of the native and esterified BF were determined with a scanning electron microscope (SEM; Quanta 200, FEI Co., Hillsboro, OR, USA), operating at an acceleration voltage of 20 kV. BF samples were mounted on circular aluminum stubs with double-sided adhesive tape and coated with gold before testing.

#### 2.3.6. X-ray Diffraction Analysis

Native and esterified BF were further dried in a vacuum oven at 50 °C for 48 h to remove the remaining moisture. Sample crystallinity indices were measured while using an X-ray diffractometer (XRD; XD-2, Beijing Purkinje General Instrument Co., Ltd., Beijing, China) with a Cu target at 36 kV and 20 mA. Samples were tested in the angular range of 2*θ* = 5–40° with a scanning rate of 4°/min. The empirical crystallization index *Crl*, proposed by Segal (Segal et al*.*, 1959), is a measure of natural cellulose crystallinity [15]. The calculation for this is

(4) $$Crl=\frac{I_{002}-I_{amorph}}{I_{002}}\times 100\%\;$$

where *I_002_* is the maximum diffraction peak intensity of the main crystallization peak 002 and *I_amorph_* the diffraction intensity of 2*θ* angles to 18°.

#### 2.3.7. Thermogravimetric Analysis

Thermogravimetric analytical (TGA) measurements of native and esterified BF were performed using a 209 F3 TGA instrument (Netzsch Instruments Inc., Burlington, MA, USA). About 5 mg of dried sample powders were placed in a platinum crucible and heated from 30 to 600 °C at the rate of 10 °C/min. Nitrogen dynamic carrier gas was applied at 30 mL/min.

## 3. Results and Discussion

### 3.1. In Situ Solid Phase Polymerization Confirmation

**Figure 3:**
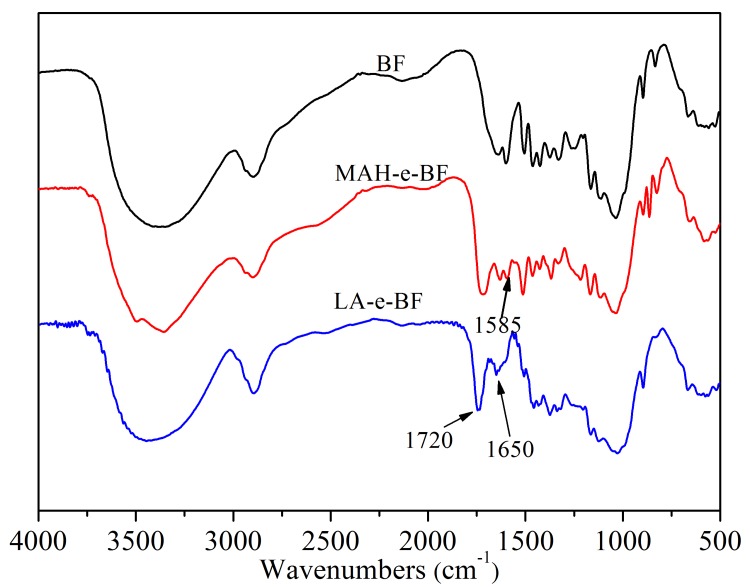
Infrared spectrogram of BF, Maleic anhydride-esterified-bamboo flour (MAH-e-BF), and lactic acid-esterified bamboo flour (LA-e-BF).

Esterification reactions between the esterifying agents and BF involved the hydrophilic hydroxyl group (-OH) in BF being replaced by the hydrophobic modifying groups (Figure 1). After esterification with MAH, BF molecules were connected to C=O and C=C, and after the esterification with LA, BF molecules were connected to C=O. FT-IR analyses of native and esterified BF were performed to verify that esterification had occurred and to investigate the resulting chemical changes (Figure 3).

In unmodified BF, its basic compositional unit is *D*-anhydroglucose, of which the main characteristic functional groups were C_2_ and C_3_-linked secondary hydroxyls and C_6_-linked primary hydroxyls and *D*-pyranose ring structures. The absorption peaks for these main structures are shown in Figure 3. The characteristic peak centered at 3310 cm^−1^ corresponded to O-H stretching and vibration of hydrogen bond associations, 2930 cm^−1^ to C-H asymmetrical stretching and vibration, 1635 cm^−1^ from the water tightly bound to the starch, 1152 cm^−1^ from C-O-C asymmetrical stretching and vibration, 1080 cm^−1^ assigned to *D*-glucopyranose and hydroxyl-linked C-O stretching and vibration, and 925 cm^−1^ due to glucosidic bond vibration. In the infrared spectrum of MAH-e-BF, in addition to all of the characteristic absorption peaks of the BF, the C=O absorption peak appeared at 1720 cm^−1^ [16,17] and the C=C absorption peak appeared at 1585 cm^−1^ [18]. LA-e-BF also showed a C=O absorption peak at 1720 cm^−1^. Following esterification with a BF-esterifying agent, unreacted MAH and LA and oligomer were removed after washing with acetone. This result confirmed that MAH and LA molecular chains were detected in the BF skeleton, thus verifying that esterification had occurred between the BF and MAH or LA. The solution titration results established that the degrees of substitution of MAH-e-BF and LA-e-BF were 21.04 ± 0.23% and 14.28 ± 0.17%, respectively.

### 3.2. Morphology Change of Esterified BF

**Figure 4:**
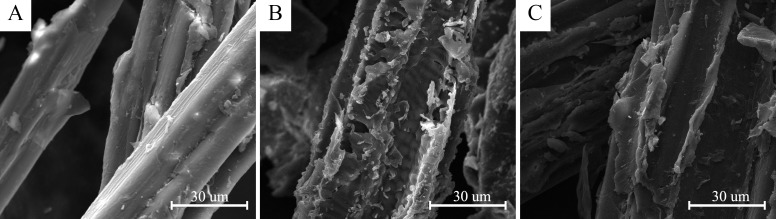
Scanning electron microscope (SEM) images of BF (**A**), MAH-e-BF (**B**), and LA-e-BF (**C**).

SEM, in principle, uses a very fine focused high-energy electron beam to scan a sample and to stimulate and collect a variety of physical information, by accepting, amplifying, and displaying this information, and the surface morphology of a test specimen is observed. The surface morphology changes of native BF, MAH-e-BF, and LA-e-BF were observed by SEM to study the extent of changes in BF surface morphology as a result of the esterification reactions (Figure 4).

The surface of native BF was smooth with few trenches, few edges, and few corners (Figure 4). When compared with BF, esterified BF surfaces were fragmentary, rough, angular, and convex. Moreover, the covering material produced by these reactions were clearly observed on these surfaces, with the surface roughness and bonding degree of MAH-e-BF clearly greater than that of LA-e-BF. These results also indicated that BF surface roughness was positively correlated with the DS. SEM test results further showed that MAH and LA were successfully reacted with BF by this in situ solid-phase esterification method, with the resulting effects from MAH-modification appearing to be better than with LA.

### 3.3. Water Resistance of Esterified BF

**Figure 5:**
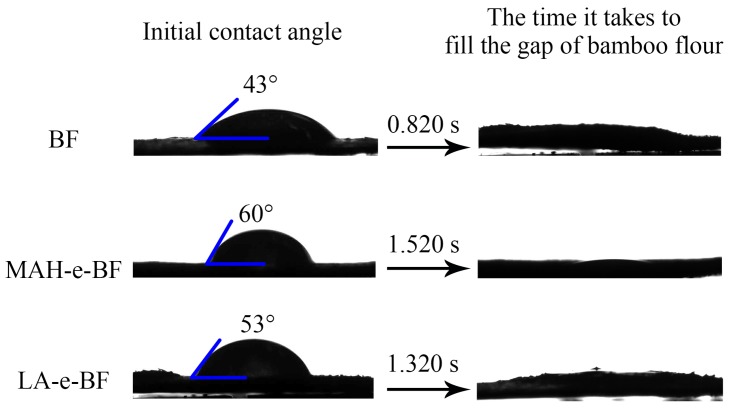
The contact angle of BF, MAH-e-BF, and LA-e-BF.

The BF molecular chain contains hydrophilic hydroxyl groups, which exhibit hydrophilic properties [19]. In this experiment, in situ solid-phase esterification of MAH and LA replaced hydroxyl groups on BF with hydrophobic groups, resulted in a reduction of the number of hydrophilic hydroxyl groups and concomitant increase in the number of hydrophobic groups, thus enhancing BF hydrophobicity. This phenomenon was verified by analyzing MAH-e-BF and LA-e-BF hydrophobicity using contact angle measurements. The water contact angle on a surface is the angle formed by a tangent line from the water droplet to a solid surface, which is an indication of the relative sample-surface hydrophobic character. The larger the contact angle, the higher the material’s hydrophobicity [20]. Native BF, MAH-e-BF, and LA-e-BF were tested while using a contact angle tester (Figure 5).

The initial contact angle of the BF was only 43° and absorbing of water droplets completely required only 0.820 s. After in situ modification by solid phase esterification, the initial contact angle increased and the full absorption time of water droplets was prolonged for both MAH-e-BF and LA-e-BF (Figure 5). The results suggested improved hydrophobicity in the esterified BF when compared to native BF. The reasons for this phenomenon included the replacement of hydrophilic hydroxyl groups on BF with hydrophobic groups, resulting the hydrophobic properties of modified BF clearly improving. SEM analysis shown that, after BF esterification with MAH or LA, BF surfaces were coated to a certain extent, reducing their water absorption capacity. MAH-e-BF exhibited a greater contact angle and longer absorption time when compared to those of LA-e-BF, indicating that MAH-e-BF hydrophobicity was better than LA-e-BF. The hydrophobicity of esterified BF was directly related to the number of hydrophobic groups that were grafted to BF and more hydrophobic groups resulted in better hydrophobicity. Thus, the measured contact angles were consistent with the DS. This was in line with the results form SEM analyses.

**Figure 6:**
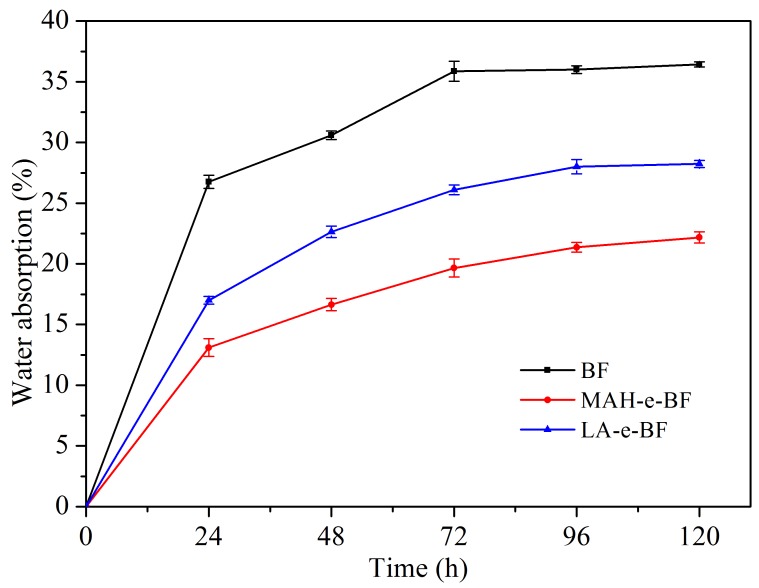
Scatter diagram of water absorption change of BF, MAH-e-BF, and LA-e-BF.

Improvement in BF hydrophobic properties of BF by ester-modification was confirmed by determining the water absorption of BF, MAH-e-BF, and LA-e-BF based on their relative weight change after exposure to water (Figure 6). Native BF water absorption gradually increased over time, but water absorption by MAH-e-BF and LA-e-BF were both lower than that of BF during soaking for 120 h, which further demonstrated that the esterification significantly improved the water resistance in modified BF. Comparison of water absorption for the two esterified BF revealed that MAH-e-BF was lower than LA-e-BF. Thus, the hydrophobicity of MAH-e-BF was found to be better than that of LA-e-BF, which was in agreement with contact angle test results.

### 3.4. Crystalline Structural Changes of Esterified BF

**Figure 7:**
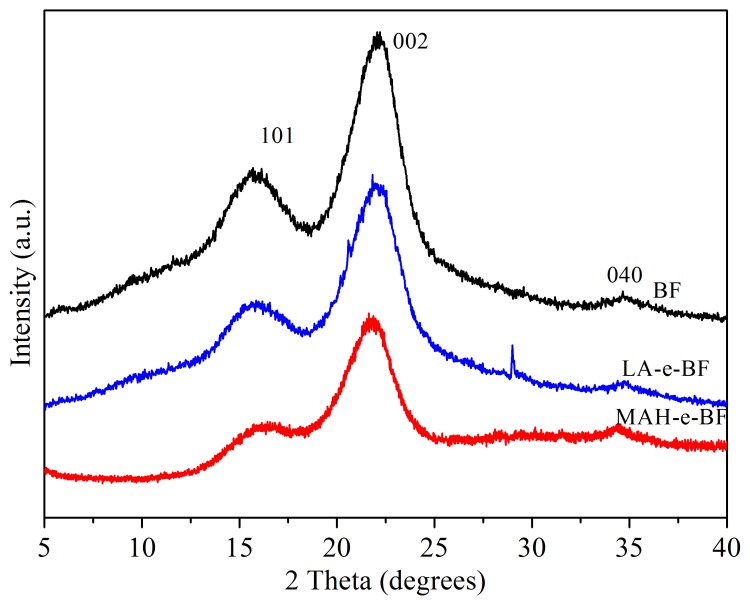
X-ray diffraction pattern of BF, MAH-e-BF, and LA-e-BF.

As BF crystal structure was easily affected by high temperatures and reactions with an esterifying agent, the crystal structure of BF, MAH-e-BF, and LA-e-BF were analyzed by XRD (Figure 7). XRD diffraction peaks for native BF were a typical of Iβ type crystalline structures, whose 2*θ* values were 16.25, 22.47, and 33.85°, which corresponded to diffraction peaks of the 101, 002, and 040 crystal faces, respectively [21]. After esterification modification, the diffraction peaks of the main crystal faces 101, 002, and 040 of MAH-e-BF and LA-e-BF were similar to those of BF. This result showed that esterification mainly occurred in noncrystalline regions of BF, because the changes in the materials’ crystalline region were very small. However, XRD diffraction peak intensities from MAH-e-BF and LA-e-BF were clearly weaker than those of native BF. Due to the effect of heat and pressure in the hydrothermal reactor, a part of the modifier will cause swelling in the crystalline region. Therefore, a part of the reaction occurs in the crystalline region, resulting in a decrease in crystallinity.

The BF crystallinity degree was calculated to be 56.78%, with the crystallinity of MAH-e-BF and LA-e-BF found to be 47.45 and 51.07%, respectively, which showed that BF crystallinity decreased after in situ solid phase esterification with MAH or LA. Because of this treatment, MAH and LA infiltrated into the crystalline area and destroyed the hydrogen bonding between molecules in the crystalline region. At the same time, BF hydroxyl groups chain-reacted with MAH or LA molecules and molecular chains gradually grew and crosslinked, which further destroyed BF crystallinity. Conversely, the destruction of the crystalline zone facilitated these reactions, which led to a further decrease in crystallinity. As BF crystallinity decreased, the forces between BF molecules were weakened [22], such that the thermal plasticity of MAH-e-BF and LA-e-BF improved. MAH-e-BF crystallinity was less than that of LA-e-BF, owing to higher DS in MAH-e-BF and more reacted BF chain hydroxyl groups, producing more serious destruction of hydrogen bonds.

### 3.5. Thermal Performance Analysis

**Figure 8:**
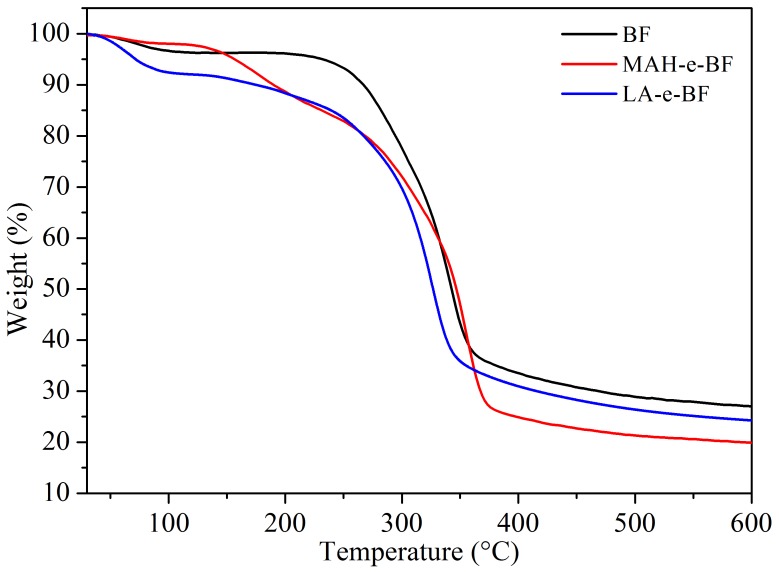
Thermogravimetric analytical (TGA) curves of BF, MAH-e-BF, and LA-e-BF.

Based on XRD analysis of esterified BF, esterification were observed to decrease BF crystallinity by changing the crystalline structure, which inevitably affected the BF thermal properties. Therefore, TGA of the material was used to determine thermal property changes in BF when it was altered to form MAH-e-BF and LA-e-BF (Figure 8). Thermal degradation of native and esterified BF was divided into three stages, with temperature ranges of 50–120, 120–400, and 400–600 °C (Figure 8). The first stage represented water evaporation from BF. In the second stage, hemicellulose, cellulose, and some xylem in BF thermally decomposed and the fastest decomposition rate was observed. In the third stage (> 400 °C), the remaining material decomposed to carbon through broken chain pyrolysis. The initiation temperatures of thermal decomposition in MAH-e-BF and LA-e-BF were clearly lower than BF and the residual ratio also lower than BF. These results were attributed to decreased crystallinity of MAH-e-BF and LA-e-BF by esterification, so that the molecular chain arrangement of cellulose in bamboo flour was reduced. This result also suggested an increase in BF plasticity as a result of esterification. When compared to MAH-e-BF and LA-e-BF, the thermal decomposition temperature and residual ratio of MAH-e-BF were lower than those of LA-e-BF, which was due to their crystallinity degrees. This also indicated that MAH-e-BF molecules were more loosely arranged and thus had better thermal plasticity.

## 4. Conclusions

This study demonstrated the successful preparation of MAH-e-BF and LA-e-BF while using an in situ solid phase esterification method, achieving DS values at 21.04 ± 23% and 14.28 ± 0.17%, respectively. Esterification resulted in the replacement of hydroxyl groups on BF *D*-anhydroglucose moieties with hydrophobic groups from MAH or LA, which improved BF hydrophobic characteristics. Esterified BF hydrophobicity was significantly higher than native BF and MAH-e-BF hydrophobicity was better than LA-e-BF. Esterified BF surfaces were covered with graft polymer, with the surface roughness and bonding degree of MAH-e-BF being clearly greater than those of LA-e-BF. The crystalline structure the esterified BF showed some damage, with the MAH-e-BF exhibiting a greater decrease in crystallinity than LA-e-BF. Overall, esterification improved BF thermoplasticity and MAH-e-BF thermoplasticity was better than LA-e-BF.

Esterified BF produced by in situ solid phase esterification exhibited increased overall hydrophobicity with concurrent increased interface compatibility, allowing for an expanded range of applications in bamboo plastic composites. This work compared the influence of two esterifying agents on BF esterification degree and hydrophobic character, providing reference data for the preparation of blended composites of BF and other polymers.
